# Editorial: Neuroendocrine Control of Feeding Behavior

**DOI:** 10.3389/fendo.2019.00399

**Published:** 2019-06-19

**Authors:** Serge H. Luquet, Hubert Vaudry, Riccarda Granata

**Affiliations:** ^1^Paris Diderot University, Paris, France; ^2^Université de Rouen, Mont-Saint-Aignan, France; ^3^Department of Medical Sciences, University of Turin, Turin, Italy

**Keywords:** energy homeostasis, appetite, satiety, nutrient sensing, orexigenic and anorexigenic neuropeptides, hypothalamus

Obesity and metabolic disorders represent major worldwide health threats. Neuroendocrine mechanisms, which play a pivotal role in the integration of hunger and satiety signals, and the regulation of energy homeostasis, holds the promises of new strategies for treatment of feeding-related diseases. This Research Topic compiles a series of review and research articles that provide a broad view of the current knowledge on the complex neuroendocrine control of feeding behavior and energy expenditure, and highlights new concepts in the field.

The hypothalamus is a key region of the brain regulating food intake and energy balance. Interestingly, deregulation of feeding behavior, causing weight loss or obesity, has been associated with high- or low-grade hypothalamic inflammation, respectively (1, 2). In their review, Le Thuc et al. focus on hypothalamic inflammation, with special emphasis on how chemokines can influence, at the hypothalamic level, the deregulation of energy balance and body weight. Indeed, in addition to being essential mediators of the inflammatory response, chemokines exert important roles at the central level by activating and attracting cells of the immune system, regulating neuronal survival and death, and also modulating the activity of certain neurons (3). Therefore, the chemokinergic system could be responsible for the deregulation of feeding behavior associated with inflammation, in both appetite and weight loss, and the development of obesity.

In relation to obesity, it has been shown that individual differences in neurobiological mechanisms controlling food intake may explain why some individuals are more susceptible to weight gain than others (4). Interestingly, one of these mechanisms is impulsivity, generally considered as the tendency to act rapidly without full consideration of consequences (5). In this respect, Michaud et al. provide a comprehensive review on alterations related to impulsivity in obesity and addiction, considering results from the personality, neurocognitive, brain imaging, and clinical fields. Overall, this review provides an approach to understand the association between obesity and addictive behaviors and, on these bases, suggests therapeutic interventions for prevention and treatment of obesity.

Obesity and diabetes have been also linked to cognitive dysfunction (6). In her review, Small investigates the mechanisms underlying the association between obesity and diabetes, and cognitive impairments and brain dysfunction, which at present are still unknown. Although studies have shown integrity of the dopamine (DA) system in cognitive dysfunction associated with diabetes and obesity, a critical role for DA adaptation in response to diet, adiposity and metabolic dysfunction has been proposed, which may explain the neurocognitive impairment observed in diabetes and obesity. However, the mechanisms underlying the involvement of the DA system in these effects remain to be clarified and will be the focus of future research.

In their study, Hankir et al. investigated whether alterations in fat appetites after Roux-en-Y gastric bypass (RYGB) associate with variations in brain μ-opioid receptors (MORs). Results from diet-induced obese male rats, undergoing RYGB, show an association between suppression of appetite, at a stage of weight loss after RYGB, and reduction of MORs, suggesting that the reduction in MOR signaling may contribute to sustained weight loss.

In relation to appetite and obesity, Santiago and Hallschmid investigated the effect of intranasal administration of insulin before sleep in healthy young and elderly humans on eating behavior in the subsequent morning. In fact, it has been shown that intranasal administration of insulin in humans reduces food intake, independently of its glucoregulatory action (7). The results of this study show that intranasal insulin administration before nocturnal sleep induces a reduction in breakfast intake in healthy subjects, with no change in energy expenditure, suggesting that, depending on sleep period, insulin may display beneficial metabolic effects and even treat or prevent insulin resistance in the brain.

Glucocorticoid hormones (GCs) are essential in the regulation of glucose and fatty acid metabolism, as well as appetite. Conditions of excess or deficiency of GC levels, such as those encountered in Cushing's syndrome or Addison's disease, respectively, are associated with severe metabolic alterations (8). Moisan and Castanon review the role of corticosteroid-binding globulin (CBG) in the regulation of GC levels. In addition to studies demonstrating the importance of CBG in influencing genetic variability of plasma GC levels, a link between CBG levels and body composition/insulin levels has also been indicated (9). Overall, recent studies have proposed a role for CBG in metabolic disorders associated with impairment of GC levels.

Ghrelin is a 28-amino acid acylated peptide produced mainly in the stomach, which has the ability to stimulate growth hormone secretion; in addition, ghrelin stimulates feeding, adiposity, and weight-gain (10). Different mechanisms are involved in the regulation of ghrelin production and signaling. It has been recently demonstrated that circulating ghrelin is in part protected from degradation by binding to immunoglobulins (Ig) (11). The review by Fetissov et al. summarizes the results on acylated ghrelin (AG) and des-acyl ghrelin (DAG) reactive Ig in conditions of altered appetite and energy balance. Overall, the authors suggest that Ig display a role in modulating the biological activities of ghrelin, including those in conditions of altered energy balance, and propose the existence of a functional link between Ig and ghrelin.

In addition to AG, the ghrelin gene-derived peptides include DAG, the most abundant form in the plasma, which binds to a yet unknown receptors and is devoid of endocrine activities, and obestatin, which displays metabolic effects but whose functions and binding characteristics are still to be fully determined (10, 12). Due to the absence of reliable assays to measure all three peptides, in their study, Hassouna et al. developed selective immunoassays combined with a highly sensitive targeted mass spectrometry method to measure and characterize the ratios of the different preproghrelin-derived peptides in mice. To validate their analyses, a preproghrelin deficient mice, that does not produce any of the peptides, was used as negative control. The results show that both forms of ghrelin and obestatin can be detected in the gastrointestinal tract of mice. Interestingly, in this organ, obestatin was found to be far less abundant than AG and DAG, likely because of reduced processing rate of preproghrelin into obestatin, or degradation of the peptide itself. It remains to be established if the main source of obestatin or its processing is outside of the gastrointestinal tract.

The hypothalamus-brainstem circuit defines a fundamental neural substrate that integrates circulating signals of hunger and satiety, nutrient, and hormones to promote adaptive behavioral and metabolic response to changes in energy demands. In order to access neural circuits these signals have to cross the blood brain barrier (BBB) (13, 14). Haddad-Tóvolli et al. provide an extensive review covering the major advances in the understanding of the BBB structure with a specific focus on hypothalamic areas. They discuss how structural adaptation of the BBB and neighboring cells such as endothelial cells, astrocytes, pericytes, tanycyte, and microglia orchestrate the regulated passage of signals from blood to brain. They also call our attention on how nutrient overload might damage the integrity and functionality of the BBB, and in turn affect neural processing of peripheral signals and appropriate control of energy homeostasis.

In the same line several contributions draft important observations regarding brain nutrient sensing. Indeed, in addition to providing energy to brain cells through their catabolism, nutrients can also be detected directly and can act as hormonal-like signals to regulate neuron and glial cell activity (15–18). Nutrient overload during consumption of energy-rich diet (fat and sugar) or in the context of obesity is associated with brain inflammatory response (19). However, it is unclear whether nutrient can directly target brain cells to create adaptive responses. Belegri et al. compared the consequence of free choice access to high-fat high-sugar diet (fcHFHS) and brain-specific infusion of lipid emulsion on endoplasmic reticulum (ER) stress and unfolded protein response (UPR) in the hypothalamus. They observed that short term exposure to a fcHFHS diet, followed by food restriction induces hypothalamic ER stress in rats, a response that was achieved comparably by direct lipid—but not glucose infusion in the brain, suggesting a direct contribution of brain lipid-sensing in that processes. This work, together with other advances in the field over the last decade, has promoted the concept that lipids entering the brain can affect neural activity and regulate, to the same extend as hormones, neural output. Bruce et al. provide an expert overview on lipid processing in the brain and cover several aspects of the mechanism by which neuron lipid sensing can result in the control of energy homeostasis. They specifically draw our attention on physiological processes by which hypothalamic neurons can detect circulating fatty acids and orchestrate the synthesis and release of triglyceride (TG) rich particles by the liver through the modulation of the autonomic nervous system outflow.

Indeed, circulating TGs represent a major source of lipid-substrate for metabolically active tissues such as heart and muscle. TGs typically increase after a meal and are packaged as chylomicrons released by the digestive tract (20). TGs also are part of liver-born lipoproteins including very-low or low density lipoproteins (VLDL, LDL). Lipoprotein lipase (Lpl) is the rate limiting enzyme for the catabolism of TGs into free fatty acids. Lpl is actively expressed in the brain, suggesting a role for brain TG sensing (21). Laperrousaz et al. elegantly show that hypothalamic Lpl is an important mediator of brain adaptive response to cold and thermogenesis. Using hypothalamic-specific invalidation approaches they demonstrate that mice lacking LPL in the hypothalamic region show increase energy expenditure and conserved body temperature. This work points out that Lpl-mediated TG-sensing in lipid-sensing neurons contribute to central regulation of thermogenesis.

Aside of lipid-sensing neurons, the hypothalamus also contains glucose-activated and glucose-inhibited neurons. The ability of these neurons to respond to glucose is an important mechanism in hypothalamic control of energy homeostasis (22). Among the different mechanisms involved in glucose sensing, Kohno et al. call our attention on sweet taste receptor-mediated glucose sensing. Sweet taste receptors are composed of heterodimers of taste type1 receptor2 (T1R2) and taste type1 receptor3 (T1R3). These receptors are widely distributed in various organs including the hypothalamus. The authors used calcium imaging to probe sweet-taste receptor signaling in arcuate neurons. They demonstrate that artificial sweeteners such as sucralose can trigger calcium response mostly in non-POMC neurons. They point to an important mechanism by which artificial sweeteners, that are widely abundant in modern food environment, may potentially alter brain-nutrient sensing and energy homeostasis.

Finally, among nutrients, amino acids (AA) are also known to signal in neural substrate regulating energy homeostasis (17). Nutritional protein input together with AA quality have great influence on metabolism and behavior. Several redundant mechanisms have evolved to insure proper balance in AA quality and quantity. Heeley and Blouet review the literature pertaining to brain AA sensing mechanisms and neural coupling to adaptive behavior. Notably, the authors highlight how bidirectional changes in essential AA availability are detected through mTOR and general control non-derepressible 2 (GCN2) pathway to adapt feeding behavior and tropism.

Several elaborated strategies are in place to optimize food consumption. Food intake responds to energy demands and metabolic needs but also to reward and emotional inputs that are processed in several brain structures integrating cognitive inputs. Competing signals such as anxiety and palatable food reward are often at play in adapted strategies and decision making behavior (23, 24). In this context, Lockie et al. explored reward-driven feeding behavior in mice in an anxiogenic context. They show that food-deprivation or ghrelin injection used as a proxy of hunger signal increase food-reward seeking and consumption in anxiogenic environment while glucose injection or *ad libitum* feeding reduce it. This works highlight how metabolic signal/nutrient can influence the assessment of safety in food-reward seeking in a risky environment. Reward encoding depends on dopamine release in the mesolimbic system (MCL) (24). The gut-brain axis has emerged as one key pillar of energy homeostasis (25). The role of the gut microbiota has particularly drawn much attention in the recent years as potentially holding important keys in whole-body homeostasis (26, 27). While microbiota manipulation can be achieved through prebiotic supplementation and has readily impact on body weight control, it remains unclear whether reward-driven components can be manipulated through digestive fibers consumption. Delbès et al. examined this aspect and show that prebiotic supplementation affects various components of food reward seeking behavior, gut microbiota ecosystem and molecular adaptation in both hypothalamic and mesolimbic structures. They found that energy-rich diet and probiotic supplementation can exert synergistic action on food reward seeking behavior and brain expression of neuropeptides involved in the regulation of body weight homeostasis. Both the nature of the diet (regular chow or HFHS) as well as the timing at which prebiotic supplementation is introduced over the course of obesogenic diet exposure greatly influence the molecular and behavioral changes underlying reward-driven behavior.

While overfeeding represents one common hallmark of obesity in modern food environment, anorexia nervosa lies at the opposite site of the spectrum and represents a devastating eating disorder whose mechanism is still largely undefined, partly due to the lack of animal model for anorexia (28). Activity-based anorexia (ABA) is a model of anorexia-like body weight loss and decreased feeding in which animals are subjected to running wheel while giving restricted-time scheduled access to food. Using that model Scharner et al. demonstrate that while female rats undergoing ABA protocol do not show alteration of short-term meal ultrastructure, brain c-fos analysis (as a proxy for neuronal activation) reveals important differences between *ad libidum* and ABA animals characterized by increased c-fos signal in brain structures controlling energy homeostasis such as the arcuate nucleus, supraoptic nucleus, locus coeruleus (LC) and nucleus of the solitary tract. This work provides an important insight into one of the rare models of body weight loss although, as pointed by the authors, this model does not fully recapitulate human anorexia as animals do not voluntarily reduce nutrient intake.

Comparative studies conducted in distant species can reveal fundamental regulatory mechanisms that have exerted strong evolutionary pressure. Teleost fish, that diverged from the mammalian lineage about 450 MYA, represent very suitable models in which to identify conserved neuroendocrine systems involved in the control of food intake and energy expenditure (29). Delgado et al. review the literature concerning the action of various neuropeptides including proopiomelanocortin (POMC), neuropeptide Y (NPY), agouti-related peptide (AgRP), cocaine- and amphetamine-regulated transcript (CART), orexin, cholecystokinin (CCK) and melanin-concentrating hormone (MCH), and various hormones e.g., insulin, leptin, ghrelin, and glucagon-like peptide 1 (GLP-1). They also discuss their implication in the hypothalamic integration of metabolic information that elicits a coordinated feeding response in fish. In spite of discrete species variations, most of these regulatory mechanisms have been highly conserved from fish to mammals.

In a sister review, Volkoff reminds us that about 30 neuropeptides are potentially involved in the regulation of feeding in fish. In addition to those aforementioned, thyrotropin-releasing hormone (TRH), orexin, galanin, apelin, and secretoneurin exert orexigenic effects whereas gonadotropin-releasing hormone 2 (GnRH2), prolactin-releasing peptide (PrRP), corticotropin-releasing factor (CRF) and its paralogs urotensin I and urocortin 3, arginine vasotocin (AVT), pituitary adenylated cyclase-activating polypeptide (PACAP), the octadecaneuropeptide (ODN), peptide YY, amylin, RFamide-related peptide-3 (RFRP-3), and nesfatin-1 act as satiety factors.

NPY is one of the most potent orexigenic peptides in the brain of mammals (30). The sequence of frog NPY is almost identical to that of the human peptide (31) and, in frog tadpoles as in rodents, intracerebroventricular (icv) injection of NPY stimulates feeding behavior (32). Matsuda et al. have investigated the downstream mechanisms through which NPY exerts its orexigenic activity in bullfrog larvae. They show that the stimulatory effect of NPY on food intake is abolished by co-administration of the selective orexin receptor antagonist SB334867. These data indicate that, in premetamorphic larvae, the orexigenic effect of NPY is mediated via the orexin/orexin receptor system.

Owing to the economic importance of poultry production, the neuroendocrine control of feeding behavior has been extensively studied in birds, notably in neonatal chicks and young chickens (33, 34). Tachibana and Tsutsui summarize the effects of various hormones and neuropeptides on feeding in birds, and highlight a few differences with what is known in mammals. For instance, ghrelin which is a potent orexigenic peptide in mammals (35) inhibits food intake in chickens (36). Reciprocally, PrPR which exerts an anorexigenic effect in mammals (37) stimulates feeding behavior in neonatal chicks (38).

Gonadotropin-inhibitory hormone (GnIH; also called RFRP3) is a member of the RFamide family of neuropeptides. The inhibitory effect of GnIH on the hypothalamo-pituitary-gonadal axis has been initially discovered in birds (39) and subsequently confirmed in fish and in mammals (40). Since energy homeostasis and reproduction are intimately correlated (41), Tsutsui and Ubuka review the evidence that GnIH exerts a coordinate control on feeding and reproductive behaviors in vertebrates.

26RFa/QRFP, another member of the RFamide family of neuropeptides, is the natural ligand of GPR103 now renamed QRFPR (42). Functional characterization of 26RFa/QFRP has revealed that *icv* injection of the peptide stimulates feeding behavior (43, 44). 26RFa/QRFP also acts on pancreatic β-cells to inhibit basal and glucose-induced insulin secretion (45, 46). Chartrel et al. review the current knowledge on the involvement of 26RFa/QRFP in the regulation of food intake and glucose homeostasis.

In a sister paper, Gesmundo et al. describe the involvement of neuroendocrine signals, including melatonin, galanin, and 26RFa/QRFP, in the control of insulin secretion. Their report highlights the key role of neurohormones in the complex regulation of β-cell activity. Together with other players, these neuroendocrine factors and their receptors represent potential therapeutic targets for the treatment of type 1 and type 2 diabetes, and metabolic disorders.

It is firmly established that the endocannabinoid system is implicated in the control of food intake and energy homeostasis (47, 48) but the underlying mechanisms have long remained unknown. Koch reviews the current knowledge on cannabinoid receptor type 1 (CB1) signaling in the regulation of feeding behavior.

Secretin, glucagon, glucagon-like peptides (GLP1 and 2), growth hormone-releasing hormone, vasoactive intestinal peptide (VIP) and PACAP belong to the same family of regulatory peptides also called the secretin family (49). Sekar et al. review the abundant literature regarding the actions of these peptide hormones and neuropeptides in the central control of feeding behavior. They point out the therapeutic potential of selective and stable analogs of these peptides, notably GLP-1 receptor agonists for the treatment of obesity and metabolic disorders.

The ventromedial nucleus (VMN) of the hypothalamus, which plays a prominent role in the regulation of food intake and energy expenditure, actively expresses the PACAP-selective receptor PAC1R (50). Consistent with this observation, microinjection of PACAP into the VMN strongly reduces food intake (51). Hurley et al. have developed a novel binge-eating model that allows to distinguish homeostatic feeding drive (hunger) from hedonic feeding drive (palatability). Their data show that injection of PACAP into the VMN decreases homeostatic feeding while injection of PACAP into the nucleus accumbens reduces hedonic feeding. These two distinct mechanisms likely contribute to the global anorexigenic effect of PACAP (52).

The central control of energy balance relies not only on neurons but also on glial cells, endothelial cells, and ependymocytes/tanycytes (53–55). Freire-Regatillo et al. review the involvement of non-neuronal cells in the transport and metabolism of hormones and nutrients participating to the neuroendocrine control of appetite and energy expenditure.

The term “endozepines” designates a family of regulatory peptides including diazepam-binging inhibitor/acyl-CoA-binding protein (DBI/ACBP) and its processing fragments, the triakontatetraneuropeptide TTN and the octadecaneuropeptide ODN, that are produced by astroglial cells and act as endogenous ligands of benzodiazepine receptors (56). Intracerebroventricular (icv) administration of ODN substantially reduces food consumption (57, 58). At the hypothalamic level, the anorexigenic effect of ODN can be ascribed to stimulation of POMC mRNA and inhibition of NPY mRNA expression (59). Guillebaud et al. here show that the DBI gene is expressed in tanycytes in the rat brainstem. Icv injection of ODN into the 4th ventricle causes a marked reduction of food intake and, in anesthetized animals, inhibits the swallowing reflex. These observations indicate that ODN acts, as an anorexigenic factor, not only within the hypothalamus, but also at the brainstem level to modify the excitability of neuronal networks implicated in feeding behavior.

DBI/ACBP belongs to the acyl-CoA-binding domain-containing protein (ACBD) family that encompasses multiple members including ACBD7 (60). Expressed in the arcuate nucleus, ACBD7 is the precursor of the non-adecaneuropeptide NDN which, like ODN, is a potent anorexigenic neuropeptide (61). Based on these observations, Lanfray and Richard describe the neurochemical mechanisms regulating the activity of ACBD7 neurons, and the downstream neuronal circuits involved in the anorexigenic effect of ODN.

There is now evidence that microRNAs (mRNAs) are involved in the central regulation of energy balance. In particular, the miRNA-processing enzyme DICER plays a pivotal role in the development, activity and survival of POMC neurons (62). Derghal et al. review the roles of miRNAs in the regulation of the melanocortin system and focus on the involvement of miRNAs in the control of POMC neurons by leptin.

The review articles and original research papers gathered in the present e-book illustrate the most recent progress in the understanding of the neuroendocrine regulation of feeding behavior and energy homeostasis. It is our hope that this Research Topic will become a major set of references for all researchers involved in this rapidly expanding field.

## Author Contributions

All authors listed have made a substantial, direct and intellectual contribution to the work, and approved it for publication.

### Conflict of Interest Statement

The authors declare that the research was conducted in the absence of any commercial or financial relationships that could be construed as a potential conflict of interest.
